# Mineralization effect of ion-releasing fiber-reinforced composite in teeth with molar–incisor hypomineralization

**DOI:** 10.2340/biid.v13.45993

**Published:** 2026-04-30

**Authors:** Lippo Lassila, Battsetseg Tseveenjav, Janna Waltimo-Sirén, Pekka Vallittu, Sufyan Garoushi

**Affiliations:** a Department of Biomaterials Science and Turku Clinical Biomaterial Center – TCBC, Institute of Dentistry, University of Turku, Turku, Finland; b Department of Pediatric Dentistry and Orthodontics, Institute of Dentistry, University of Turku, Turku, Finland; c Department of Oral and Maxillofacial Diseases, University of Helsinki and Helsinki University Hospital, Helsinki, Finland; d Department of Maxillofacial Surgery, Päijät-Häme Joint Authority for Health and Wellbeing, Päijät-Häme Central Hospital, Lahti, Finland; e The Wellbeing Services County of Southwest Finland, Turku, Finland

**Keywords:** molar–incisor hypomineralization, dentin mineral content, Fuji II LC, ion-releasing, short fiber composite

## Abstract

**Objective:**

To evaluate the long-term mineralizing effects of an experimental ion-releasing, short fiber-reinforced flowable composite (SFC-active) applied to human teeth with molar–incisor hypomineralization (MIH).

**Methods:**

A total of 16 first permanent molars, extracted due to MIH, received two occlusal restorations each. All cavities were acid-etched for 15 seconds before applying the restorative materials. One of the cavities in each tooth was restored with a commercial conventional particulate-filled composite (PFC; G-aenial Universal Injectable) after placement of the SFC-active liner. The other cavities were restored without the liner, using PFC alone (*n* = 8) or resin-modified glass ionomer cement (RMGIC; Fuji II LC) alone (*n* = 8). The teeth were stored in simulated body fluid at 37°C for 30 months. The mineralization effect was assessed at three regions (coronal, middle, and apical) under the restorations using micro-computed tomography (CT) (dentin density), micro-indentation (dentin hardness) and scanning electron microscopy/energy-dispersive spectroscopy (microstructure and calcium-to-phosphorus [Ca/P] ratio) analyses.

**Results:**

Micro-CT analyses revealed no statistically significant differences (*p* > 0.05) in dentin mineral density between the restorative materials at any of the three regions beneath the restorations. At the coronal region of interface, dentin hardness was higher with SFC-active than with PFC, but lower than with RMGIC (*p* < 0.05). The Ca/P ratios of dentin varied beneath the different restorations, ranging from 1.49 to 1.60, with the highest ratios observed at the coronal region of the interface with SFC-active. Strontium and fluorine were detected in the dentin adjacent to the RMGIC restorations.

**Conclusion:**

SFC-active demonstrated a positive mineralizing effect on dentin, reflected by higher hardness and Ca/P ratios at the coronal region of the interface. These findings indicate that SFC-active is a promising restorative material for the management of MIH-affected teeth.

## Introduction

Molar–incisor hypomineralization (MIH) is a developmental enamel defect that affects up to 13–14% of children worldwide [1]. The condition results in enamel that is porous, hypomineralized, and structurally fragile, predisposing affected teeth to post-eruptive breakdown, rapid caries progression, and hypersensitivity [2]. Although MIH is primarily an enamel defect, the underlying dentin may also display compromised mineralization and reduced strength due to increased permeability and acid challenges, which further complicate restorative outcomes. The likelihood of caries in MIH-affected teeth can be up to 4.6 times higher than in sound teeth [3], and the affected molars are particularly vulnerable to masticatory damage compared with affected incisors [4].

The management of MIH poses a major clinical challenge in pediatric dentistry [5]. Conventional restorative materials exhibit poor adhesion to hypomineralized enamel, leading to frequent restoration failures, discomfort, and the need for repeated treatment in young patients. In recent decades, clinical guidelines have been established to assist practitioners in applying scientific evidence to enhance treatment practices. These guidelines emphasize that restoring hypomineralized teeth remains particularly challenging, not only because structural defects reduce the mechanical strength of the affected tissue but also due to the demanding treatment process involving extensive restorations that are difficult to maintain in high stress-bearing areas [6, 7].

Currently available direct restorative materials, such as glass ionomer cements (GICs), compomers, and resin composites, each have significant limitations when used in MIH cases. Glass ionomers offer ion-release and chemical adhesion but lack the mechanical strength required for long-term durability in stress-bearing areas [6]. Conversely, resin composites provide good esthetics and strength but have no ion release and bond poorly to compromised enamel [8]. These limitations highlight the need for a restorative material that combines structural reinforcement, bioactivity or a mineralizing effect, bonding ability, and clinical applicability, specifically tailored for managing MIH in children. Strengthening resin composite and GIC by incorporating short glass fibers has been shown to be an effective strategy offered by materials science [9, 10]. As documented, incorporated short fibers improve the material’s resistance to crack propagation and concurrently decrease the stress intensity at the crack tip, preventing an unstable spread of cracks. Thus, the fibers increase the material’s fracture toughness and fracture resistance [11].

In recent years, commercial short fiber–reinforced composites (SFCs) have demonstrated success in restoring severely damaged teeth and managing large, high-stress-bearing cavities. Numerous laboratory studies have shown that these materials exhibit enhanced fracture toughness, crack-stopping behavior, and more favorable stress distribution under load compared with conventional direct restorative materials [12–14]. Hence, SFCs provide a promising model for developing new restorative systems that combine strength with ion-release capacity [15–17].

In our previous work, we developed and characterized a SFC-active, introducing a novel concept that combines short glass fibers with bioactive functional fillers. This experimental flowable composite demonstrated release of calcium, phosphate, zinc, titanium, and fluoride ions while maintaining superior fracture resistance and toughness compared with conventional restorative systems [18, 19]. Importantly, the material preserved both its ion-release capability and mechanical performance over time, supporting its potential clinical durability [19]. Long-term aging conditions are essential for evaluating the durability of ion release and mineralizing effects, as short-term tests often overestimate the bioactivity of restorative materials.

Building upon these findings, this study investigates whether the experimental ion-releasing SFC-active composite can promote dentin mineralization in MIH-affected teeth. SFC-active was evaluated after long-term storage in terms of dentin mineral density, hardness, and elemental composition, and compared with an inert particulate-filled composite (PFC) and a commercial ion-releasing material (resin-modified glass ionomer cement, RMGIC). The null hypothesis was that there would be no significant differences in dentin mineralization at the interface among the restorative materials.

## Materials and methods

### Materials

Bisphenol-A-glycidyl dimethacrylate (bis-GMA, purity 97%) was purchased from Esstech Inc. (Essington, PA, USA). Diurethane dimethacrylate (UDMA, purity 97.9%), triethylene glycol dimethacrylate (TEGDMA, purity 95%), camphoroquinone (CQ, purity >98%), and N,N’-dimethyl aminoethyl methacrylate (DMAEMA, purity 98–99%) were obtained from Sigma-Aldrich Co. (St Louis, MO, USA). Borosilicate glasses containing TiO_2_ and ZnO filler particles (Ø 0.7 µm) were received from Schott AG (UltraFine GM27884, Schott, Landshut, Germany). Carbonated apatite particles (Cytrans®, Ø 10 µm) and glass fibers having a length scale of 200–300 micrometer (Ø6 μm) were obtained from GC Corporation (Tokyo, Japan). Calcium carbonate powder having a primary particle size of 200 nm (purity 99.9%) was supplied by Shiraishi Central Laboratories (Hyogo, Japan). All of the reagents were used without purification.

Commercial inert flowable PFC (G-aenial Universal Injectable, GC Corporation) and RMGIC (Fuji II LC, GC Corporation) were used as controls.

### Production of ion-releasing fiber-reinforced composite

Experimental bioactive flowable SFC-active was prepared by mixing 38 wt.% of dimethacrylate-based resin matrix (bisGMA/UDMA/TEGDMA = 30/30/40 with 0.7 wt.% CQ and 0.7 wt.% DMAEMA as a photoinitiator system) to 21 wt.% of TiO_2_ and ZnO-containing borosilicate glass powder, 18 wt.% of carbonated apatite particles, 3 wt.% of calcium carbonate powder, and 20 wt.% of short fibers. The E-glass fibers (as-received silanized), so-called microfibers and non-silanated powders were added gradually to the resin matrix. The mixing was carried out by using a high-speed mixing machine for 2 minutes (Hauschild Speed Mixer DAC 400.1, 3500 rpm). Temperature change during the mixing was monitored using an infrared thermometer.

### Specimen preparation

MIH-affected teeth were collected at the Children’s Hospital of the Helsinki University Central Hospital (HUCH). Permission for the collection of extracted teeth was obtained from the City of Helsinki (HEL 2018-003492 T130201), where pediatric patients come to the HUCH by referral. Informed consent was provided, and permission from parents was sought for the use of extracted teeth for research purposes. Teeth extractions were part of the patient’s individualized routine treatment, based on pediatric dentists’ treatment decisions.

According to Finnish national regulations (law No. 101/2001, Section 20, Act on the Medical Use of Human Organs, Tissues and Cells), ethical approval is not required for research using extracted human teeth when the teeth have been removed for medical reasons. Therefore, no specific ethical approval was required for the use of the extracted teeth in this study.

**Figure 1:**
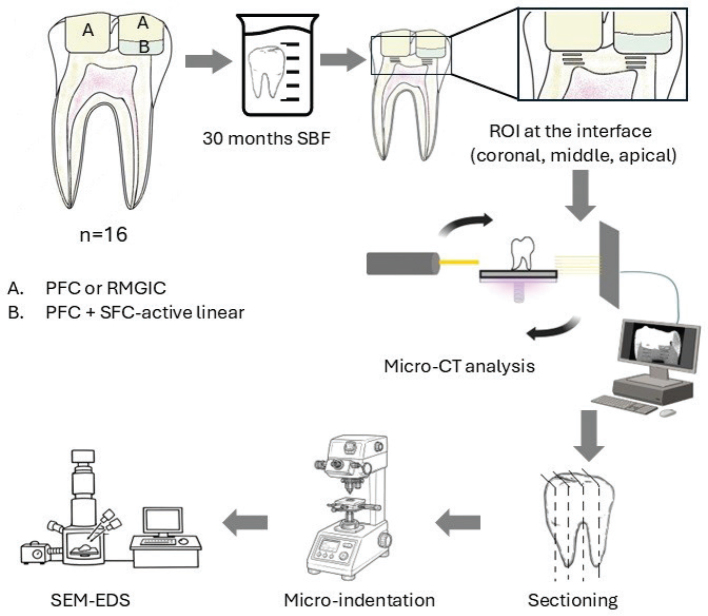
Schematic workflow of the study illustrating specimen preparation and analytical procedures for restored MIH-affected teeth. MIH: molar–incisor hypomineralization; PFC: particulate-filled composite; SBF: simulated body fluid; ROI: region of interest; RMGIC: resin-modified glass ionomer cement; SFC: short fiber-reinforced flowable composite; CT: computed tomography; SEM: scanning electron microscopy; EDS: energy-dispersive spectroscopy.

A total of 16 permanent MIH-affected first molars were collected and stored in 0.5% chloramine-T at 4°C until use. The teeth presented with caries but had no previous restorations. Straight diamond burs (835.012/836.018, Edenta AG, Switzerland) were used for cavity preparation under constant water cooling. The cavities extended deep in dentin but did not result in pulp exposure. All cavity preparations and direct restorations were performed by the same trained operator. Each tooth received two occlusal restorations. One of the cavities in each tooth was restored with the commercial conventional PFC after placement of the SFC-active liner (*n* = 16). The other cavities were restored without the liner, as controls, using PFC alone (*n* = 8) or RMGIC alone (*n* = 8) (Figure 1).

All cavities were acid-etched with 37% phosphoric acid (Scotchbond Universal Etchant, 3M, St. Paul, MN, USA) for 15 seconds, then thoroughly rinsed with water and gently air-dried prior to restoration. This protocol was selected to ensure effective and standardized enamel and dentin conditioning and to promote reliable adhesion of the applied restorative materials [20]. The SFC-active liner was applied and light-cured in a single 2-mm layer. Both the PFC and RMGIC were placed in 2-mm increments, and each layer was light-cured for 20 seconds at 1000 mW/cm² (Elipar DeepCure-L LED, 3M). The specimens were stored in simulated body fluid (SBF) at 37°C for 30 months, with the SBF renewed every 3 months. The SBF was prepared according to a well-known formula [21].

To evaluate the interface between the restorative materials and dentin after storage, three analytical methods were employed, each applied to specific regions of interest (Figure 1). Measurements were conducted at three depths (regions of interest): directly at the material–dentin interface (coronal), 1 mm below the interface (middle), and an additional 1 mm apically (apical). Micro-computed tomography (CT) was used to quantify dentin mineral density, micro-indentation was performed to assess dentin hardness, and microscopic analyses (scanning electron microscopy/energy-dispersive spectroscopy [SEM/EDS]) were carried out to examine microstructural and elemental characteristics.

### Micro-CT analysis

All teeth were scanned using a high-resolution desktop micro-CT system (SkyScan 1272, Bruker, Kontich, Belgium). Each tooth was mounted on a sample holder using transparent orthodontic utility wax to ensure stability during scanning. The scanning parameters were as follows: 70 kV source voltage, 142 μA source current, 5 μm pixel size, and a rotation step of 0.5°. Each tooth was scanned through a full 360°, producing 720 projection images. The average scan time per specimen was approximately 6 minutes.

Image reconstruction was performed using NRecon software (version 1.7.5.6, SkyScan), with InstaRecon (version 2.0.4.5, SkyScan) used to accelerate the reconstruction process. The reconstructed datasets were analyzed using CTAn software (SkyScan) for volumetric assessment. The processing workflow included global thresholding followed by despeckling to remove small artifacts.

For analysis, the entire tooth was initially selected as the general region of interest (ROI) to capture all dentin. Subsequently, the dentin was divided into three specific regions at different depths, as described earlier: coronal, middle, and apical, using anatomical landmarks along the long axis of the tooth. Within each region, a consistent ROI was defined, and the shrink-wrap tool was applied to isolate dentin from surrounding structures. Dentin mineral density was then calculated within these regional ROIs after additional rounds of thresholding and despeckling to refine the dataset. Processed data from each region were exported to an Excel spreadsheet for statistical evaluation.

### Micro-indentation

Teeth were sectioned mid-sagittally in the mesio-distal plane using a ceramic cutting disc (Struers, Glasgow, Scotland) operating at 100 rpm under water cooling. Dentin surface hardness was measured at the same three regions of interest (coronal, middle, and apical) using a Vickers micro-indentation tester (SMT-5000, Rtec Instruments, CA, USA). For each specimen, a suitable indentation area was identified under the tester’s microscope. Six indentations were performed per specimen under a 1 N load with a dwell time of 30 seconds. Indentations were spaced approximately 0.5 mm apart and positioned to avoid overlap between neighboring indents and across the different regions.

### Microscopic analysis

The microstructure of the investigated specimens was characterized using scanning electron microscopy (SEM; LEO, Oberkochen, Germany), and elemental composition was analyzed with energy-dispersive spectroscopy (EDS) at the areas of interest. Specimens were first stored in a desiccator for 24 hours, and then coated with a thin gold layer using a sputter coater in a vacuum evaporator (BAL-TEC SCD 050, Balzers, Liechtenstein) prior to SEM/EDS examination.

### Statistical analysis

The data (dentin mineral density & micro-hardness) were statistically analyzed with SPSS version 23 (SPSS, IBM Corp.) using analysis of variance (ANOVA) at the *p* < 0.05 significance level, followed by a Tukey Honestly Significant Difference (HSD) *post hoc* test to determine the differences between the groups. Levene’s test was used to assess the homoscedasticity of variances. The restoration (cavity) was considered the experimental unit. As two restorations were placed within each tooth, potential intra-tooth dependence cannot be excluded and should be considered when interpreting the results.

## Results

**Figure 2:**
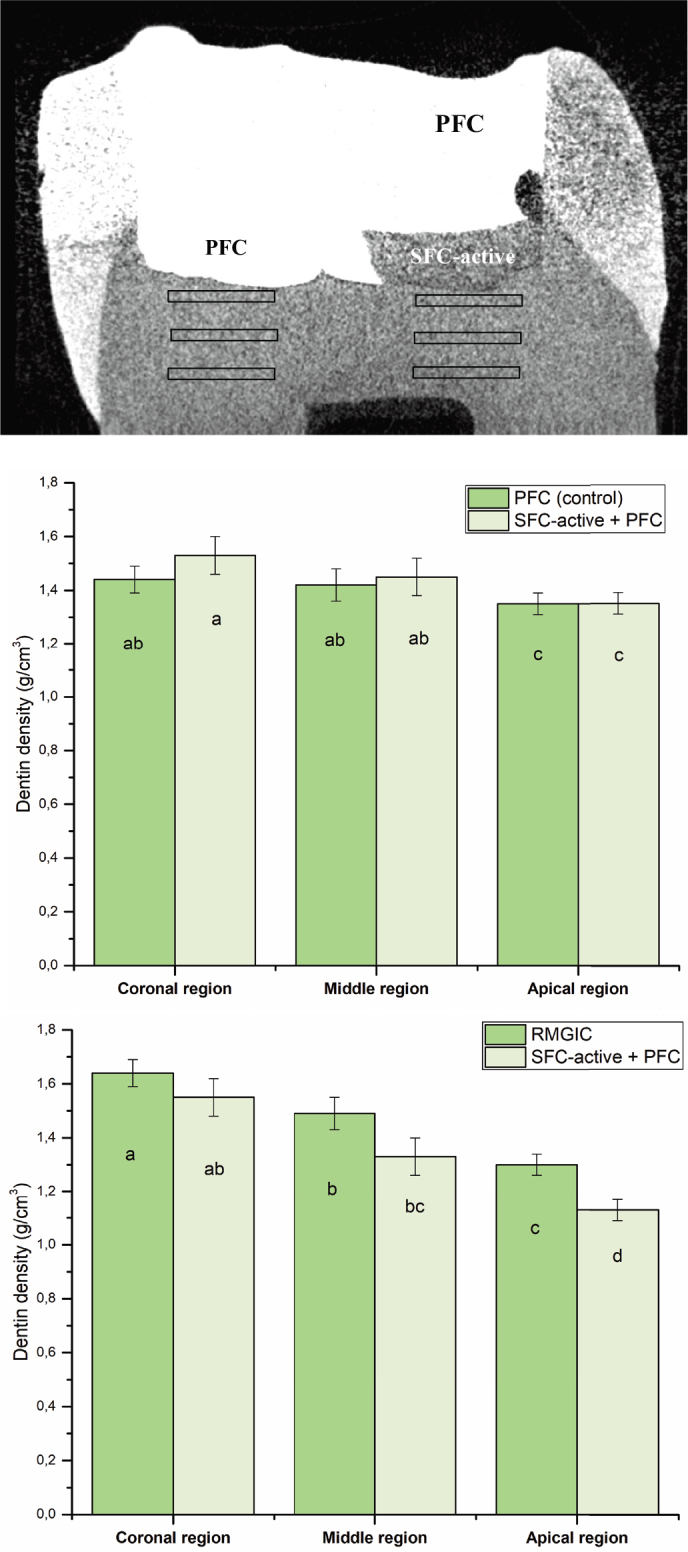
Representative cross-sectional view of the restored tooth with micro-CT, which showed the two restorations and regions of interest under the restorations. Mean dentin mineral density (g/cm³) measured beneath PFC, SFC-active, and RMGIC restorations at three regions of interest: coronal, middle, and apical. The same letters inside the bars represent non-statistically significant differences (*p* > 0.05) among the groups. PFC: particulate-filled composite; RMGIC: resin-modified glass ionomer cement; SFC: short fiber-reinforced flowable composite; CT: computed tomography.

Micro-CT analyses (Figure 2) revealed no statistically significant differences (*p* > 0.05) in dentin mineral density among the three regions beneath the restorations when different materials were compared within the same teeth. In contrast, ANOVA revealed a significant effect of region (*p* < 0.05), confirming that dentin mineral density decreased from coronal to apical regions regardless of the restorative material.

**Figure 3:**
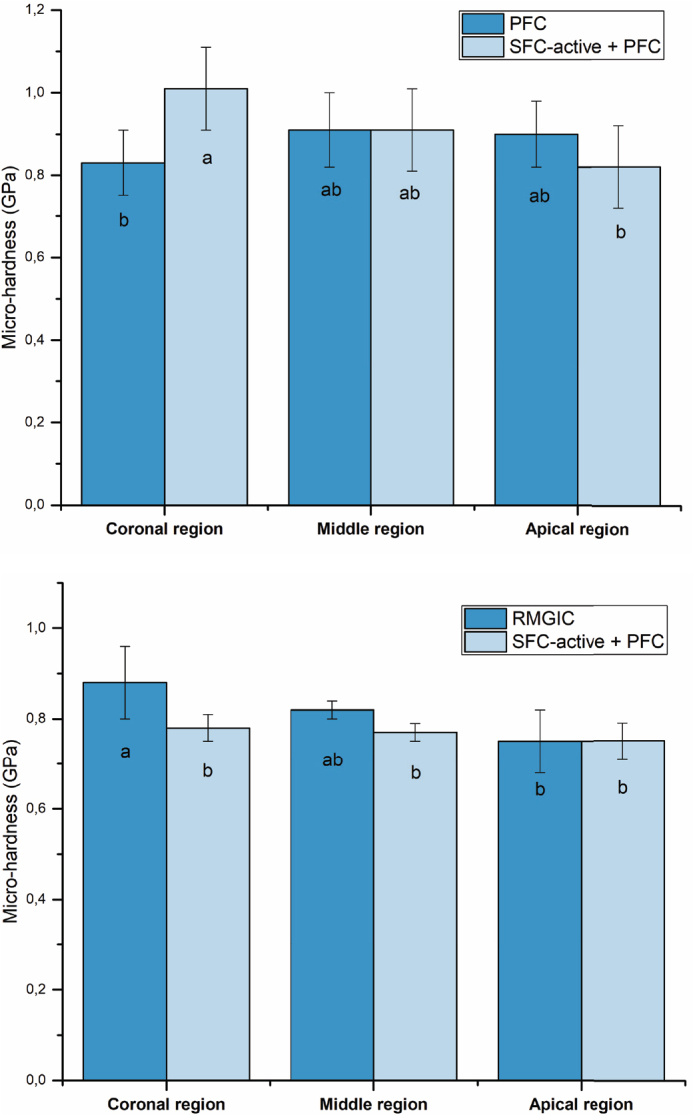
Mean dentin micro-hardness (GPa) measured beneath PFC, SFC-active, and RMGIC restorations at three regions of interest: coronal, middle, and apical. The same letters inside the bars represent non-statistically significant differences (*p* > 0.05) among the groups. PFC: particulate-filled composite; RMGIC: resin-modified glass ionomer cement; SFC: short fiber-reinforced flowable composite.

Dentin hardness at the coronal region of the interface with SFC-active (Figure 3) was significantly higher than that measured adjacent to the PFC composite (*p* < 0.05). In contrast, dentin hardness at the coronal interface adjacent to RMGIC was significantly higher than that observed under SFC-active (*p* < 0.05). However, no significant differences were detected between materials in the middle or apical regions (Figure 3).

**Figure 4:**
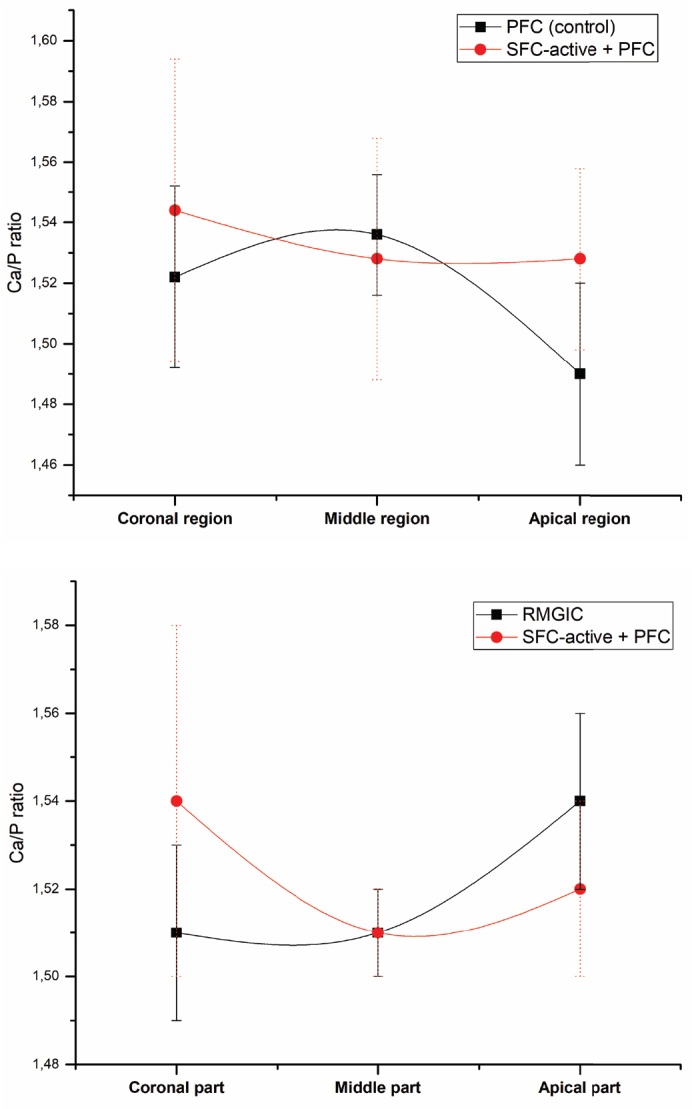
Mean dentin Ca/P ratio measured beneath PFC, SFC-active, and RMGIC restorations at three regions of interest: coronal, middle, and apical. PFC: particulate-filled composite; RMGIC: resin-modified glass ionomer cement; SFC: short fiber-reinforced flowable composite; Ca/P: calcium-to-phosphorus.

The calcium-to-phosphorus (Ca/P) ratios of dentin (Figure 4) varied among teeth restored with different materials, ranging from 1.49 to 1.60, with the highest ratios consistently observed at the coronal region of the interface with SFC-active.

**Figure 5:**
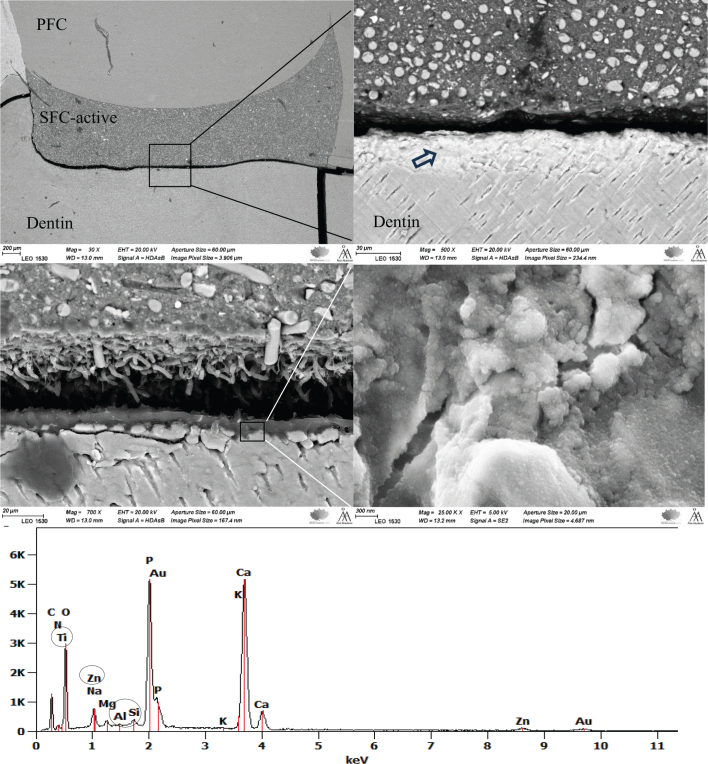
SEM/EDS images at different magnifications show signs of dentin mineralization at the interface with SFC-active. The EDS analysis was performed on the area indicated by the arrow. PFC: particulate-filled composite; SFC: short fiber-reinforced flowable composite; SEM: scanning electron microscopy; EDS: energy-dispersive spectroscopy.

SEM images (Figure 5) showed signs of mineralization in dentin at the interface (coronal region) with SFC-active, and EDS analysis revealed the presence of calcium, phosphorus, silicon, zinc, aluminum, and titanium.

Interestingly, strontium (1.66 wt.%) and fluorine (1.14 wt.%) were detected in the dentin adjacent to RMGIC restorations, reflecting their release and diffusion from the material.

**Figure 6:**
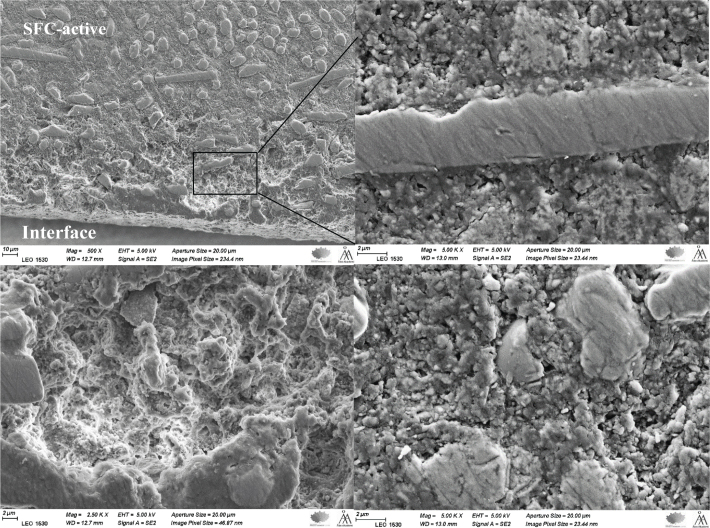
SEM images at different magnifications of the SFC-active material at the dentin interface show signs of localized matrix disruption or degradation. SFC: short fiber-reinforced flowable composite; SEM: scanning electron microscopy.

Figure 6 shows the SFC-active material in close proximity to the dentin interface. Localized areas of matrix disruption or degradation and pores were observed, which may be associated with the ion-releasing activity of the material.

## Discussion

The present study evaluated the potential of the experimental ion-releasing SFC-active to promote dentin mineralization in MIH-affected permanent molars after long-term storage in SBF. SFC-active showed trends toward higher mineralization-related parameters, particularly at the coronal region of the dentin–material interface, supporting the concept that combining short fibers with bioactive fillers may offer a promising approach for restoring structurally compromised MIH teeth. The null hypothesis was therefore partially rejected, as some differences were observed among the restorative materials.

Micro-CT analysis did not demonstrate statistically significant differences in dentin mineral density among the tested materials. However, a numerical trend was observed in the coronal dentin region, where dentin adjacent to SFC-active showed slightly higher mineral density than dentin under the conventional composite, but slightly lower than dentin adjacent to RMGIC. Although these differences were not statistically significant, the observed numerical trends may suggest a potential influence of ion-releasing materials on interfacial mineralization. This observation is consistent with previous studies reporting that bioactive restorative materials capable of releasing calcium, phosphate, and other functional ions can increase ionic saturation at the restoration–dentin interface, potentially promoting mineral deposition and stabilization of the demineralized collagen matrix [18, 22–24]. The slightly higher density observed adjacent to RMGIC may be related to its release of fluoride and strontium ions [19, 24]. In addition, significant regional differences in dentin density were observed, decreasing from the coronal to the apical region. This finding is consistent with the natural gradation in MIH lesions, where coronal dentin is typically more exposed to ion diffusion from restorative materials and to the remineralizing environment of SBF. This pattern was observed across all materials, indicating that the coronal region may be more responsive to mineralizing conditions [25].

Dentin hardness followed a similar pattern to mineral density. SFC-active significantly increased hardness at the coronal region compared with PFC, whereas RMGIC yielded significantly higher values than those observed with SFC-active. These findings suggest that the ion-release profiles of SFC-active and RMGIC create an environment promoting the hardening of relatively soft MIH dentin, potentially restoring its mechanical integrity. The absence of significant differences between materials in the middle and apical regions likely reflects limited ion penetration and a reduced mineralizing effect deeper within the tissue.

It is important to recognize that the present findings were obtained from extracted teeth in which the intrinsic dentin fluid dynamics and pulpal pressure are absent. *In vivo*, outward fluid flow from the dentinal tubules, driven by pulpal vascular pressure, may influence the diffusion and transport of ions at the material–dentin interface [26, 27]. The lack of this physiological fluid movement in the current experimental setup may therefore underestimate the extent or rate of ion exchange occurring clinically.

The Ca/P ratio is widely used as an indicator of hydroxyapatite mineral quality. In the present study, Ca/P ratios ranged from 1.49 to 1.60, slightly below the theoretical value for stoichiometric hydroxyapatite (~1.67) [28] but still within a biologically relevant range for apatite [29]. These lower ratios may reflect the pre-existing demineralization of MIH-affected teeth, which are often hypomineralized and structurally compromised by caries, suggesting an incomplete re-establishment of the stoichiometric mineral composition typical of healthy dentin at the interface with the restorative material. The mineral phase formed at the interface is therefore likely to consist of calcium-deficient or non-stoichiometric apatite, rather than fully crystalline hydroxyapatite. Such phases are commonly observed during early stages of mineralization and may also include amorphous calcium phosphate precursors, which can subsequently transform into more stable apatite structures. Although these phases differ from stoichiometric hydroxyapatite, they can still contribute to mechanical reinforcement and act as a source of calcium and phosphate ions for ongoing remineralization [30].

The highest Ca/P ratio was consistently found at the SFC-active interface (coronal region), suggesting that this material promotes the formation or retention of calcium-rich mineral phases (Figure 5). This could be explained by the high concentration of functional bioactive fillers (> 40 wt.%), in particular calcium carbonate and carbonated apatite (Cytrans®) within the composition of SFC-active. Furthermore, the presence of TEGDMA, known for its relatively high hydrophilicity, may enhance water sorption and thereby promote ion diffusion through the polymer network rather than entrapment within the crosslinked matrix [31, 32]. Previous studies have reported that materials with high filler content combined with a weak matrix–filler interface exhibit increased susceptibility to water-related degradation [33, 34]. In this context, the use of non-silanated bioactive fillers, together with the hydrophilic monomer components, likely contributes to the observed ion release behavior and supports the enhanced Ca/P ratios at the dentin interface. In addition, a recent report suggests that exposed short fibers might have a positive role in ion leaching from the cross-linked composite structure [35]. On the other hand, the amount of calcium release from RMGIC may be too low to increase the Ca/P ratio, which is in line with previous reports [36, 37].

EDS confirmed that in addition to calcium and phosphorus oxides, zinc-, and titanium oxides were present within the coronal interfacial dentin. These compounds might have inhibitory effects on biofilm formation and adhesion [38–40], an issue that will be investigated further in the near future. RMGIC displayed clear diffusion of strontium and fluorine, which is consistent with its known ion-release profile. Strontium is known to provide potential antibacterial activity [41]. The presence of these ions in dentin adjacent to RMGIC reinforces the validity of the analytical methods and highlights the difference between the two ion-releasing materials. As expected, no ion release was detected from the conventional inert PFC, which is consistent with previous reports demonstrating that particulate-filled resin composites lack bioactivity and do not contribute to ionic exchange or mineralization processes [18].

When the results are considered together, a consistent numerical trend was observed between mineral density, microhardness, and elemental composition at the coronal region of the dentin–material interface. Regions with relatively higher mineral density tended to show increased hardness and higher concentrations of calcium- and phosphate-containing phases as well as material-dependent ions. However, no statistical correlation between the methods was performed; therefore, these observations should be interpreted as descriptive trends rather than confirmed relationships.

Localized areas of disruption within the matrix of the SFC-active were noted near the material-dentin interface. This might be attributed to the resorption and release rate of the functional bioactive particles within the SFC-active structure. Calcium carbonate has shown very fast resorption and release rates in comparison with other bioactive particles [42], while carbonated apatite has shown a low resorption rate with a constant/prolonged release of calcium ions [43, 44]. In addition, such localized matrix alterations may result from hydrolytic degradation of the SFC-active polymer matrix during prolonged storage in contact with dentin. Hydrophilic resin components, such as TEGDMA, are known to absorb water during storage, which can lead to slight swelling and potential disruption of the polymer network [45]. Therefore, the observed features are likely multifactorial and should be interpreted with caution. Nevertheless, limited matrix degradation may also reflect ion exchange processes and surface reactions that facilitate mineralization, as previously described for other bioactive materials, where initial dissolution precedes mineral deposition [22].

The clinical management of MIH remains challenging due to poor adhesion, rapid material breakdown, and limited durability of conventional restorative materials. SFC-active may overcome several of these limitations by combining short-fiber reinforcement [18, 19] with the release of ions (Ca, P, Zn, Ti), which support mineralization and strengthen the interface with hypomineralized dentin. Compared with other ion-releasing restorative materials, such as bioactive composites, GICs, and calcium silicate–based materials, SFC-active offers the combined advantage of structural reinforcement and slow ion release, suggesting that it is a promising bioactive material that also maintains mechanical stability [19]. Interestingly, a recent systematic review reported that bioactive restorative materials did not provide additional clinical benefit in enhancing anticaries activity or the longevity of direct posterior restorations compared with conventional composites [46]. Furthermore, a study by Van Dijken, Pallesen, and Benetti [47] observed that one commercial bioactive composite exhibited a higher clinical failure rate than conventional composites. These findings highlight the need for further research in this area, particularly focusing on MIH-affected teeth.

Several limitations of this study should be acknowledged. First, the sample size was modest and limited by the availability of extracted molars affected by MIH, and the absence of an additional experimental group, such as using SFC-active alone without coverage. In addition, no *a priori* sample size calculation was performed; thus, statistical power may be limited. Second, the *in vitro* nature of the study does not replicate the complex biomechanical and biochemical conditions of the oral environment. Third, the mineralization effects were evaluated after long-term storage, but the dynamic processes occurring during earlier stages were not captured. Future studies should include longitudinal analyses at multiple time points, mechanical fatigue testing, and clinical trials to validate the long-term performance of SFC-active in children with MIH.

## Conclusion

Within the limitations of this study, the findings suggest that SFC-active may promote localized mineralization of dentin in MIH-affected molars. Trends toward increased interfacial mineral density and hardness, as well as ion exchange with the underlying dentin, were observed, although not all differences were statistically significant.

## Data Availability

The datasets used and/or analysed during the current study available from the corresponding author on reasonable request
